# Supplementary material for: Mechanistic insight into the chemical treatments of monolayer transition metal disulfides for photoluminescence enhancement
Source: Nat Commun. 2021 Oct 18;12:6044. doi: 10.1038/s41467-021-26340-6 (PMC8523741; doi:10.1038/s41467-021-26340-6)
Supplement: Supplementary file 1 — Supplementary Info [file 41467_2021_26340_MOESM1_ESM.pdf]

Supplementary Information

Mechanistic Insight to the Chemical Treatments of Monolayer Transition Metal Disulphides for Photoluminescence Enhancement

*Zhaojun Li, Hope Bretscher, Yunwei Zhang, Géraud Delport, James Xiao, Alpha Lee, Samuel D. Stranks, and Akshay Rao**

Contents

1. Supplementary Note 1 - experimental details 2
2. Supplementary Note 2 - calculation details 2
3. Supplementary Note 3 - PL data for chemical treated MoS_2 and WS_2 3
4. Supplementary Note 4 - Raman data for MB and F4TCNQ-treated MoS_2 14
5. Supplementary Note 5 - XPS data for pristine H-TFSI and Li-TFSI treated MoS_2..... 15
6. Supplementary Note 6 - DFT simulation data for WS_2 15
7. Supplementary Note 7 - pump-probe and TRPL spectra for MoS_2 17
8. Supplementary Note 8 - PL data for M_3-Tf and Li-OAc-treated MoS_2 and WS_2 19
9. Supplementary Note 9 - DFT simulation of anion adsorption on MoS_2 surface 20
10. Supplementary Note 10 - Raman data for M_3-Tf and Li-OAc-treated MoS_2 21
11. Supplementary Note 11 - TRPL and PL diffusion data for Li-Tf-treated MoS_2.......... 22
12. References 22

1. Supplementary Note 1 - experimental details

$Si\text{-}SiO_2$ substrates with 90 nm oxide layer were used for steady-state photoluminescence (PL), Raman spectroscopy and X-ray photoemission spectroscopy (XPS). Quartz substrates were used for time-resolved photoluminescence (TRPL), ultrafast pump-probe measurement, and PL diffusion measurements. The samples were encapsulated for ultrafast pump-probe measurements, and other measurements are carried out on samples without encapsulation.

2. Supplementary Note 2 - calculation details

First-principle calculations of formation energies were carried out based on the density functional theory (DFT) with the Perdew-Burke-Ernerhof (PBE) exchange-correlation functional as implemented in the VASP code. The all-electron projector-augmented wave (PAW) method was adopted, where $4d^5 5s^1$, $5d^4 6s^2$, $3s^2 3p^4$, $1s^1$, $2s^1$, and $3s^1$ are treated as valence electrons for Mo, W, S, H, Li and Na atoms, respectively. The plane-wave energy cutoff is set to 600 eV. A Monkhorst-Pack Brillouin zone sampling grid with a resolution of $2\pi \times 0.03$ Å^{-1} is adopted to ensure that all the enthalpy calculations are well converged with an error less than 1 meV/atom. Structural relaxations were performed with forces converged to less than 0.01 eV Å^{-1}. The 3×3 hexagonal supercell of monolayer MoS_2 and WS_2 were utilized to display various available adsorption sites in MoS_2/ WS_2 for M_1 (H, Li, Na) adsorption. A vacuum spacing of 20 Å was provided along a perpendicular direction to the plane of MoS_2/ WS_2 between two adjacent periodic layers in order to avoid any spurious interactions. Detailed structure information is listed below.

Taking MoS_2 as the example, the stability of various adsorption sites is calculated from their formation energy, which is defined as:

$$E = E_{M1+MoS_2} - E_{MoS_2} - E_{M1} \#(S1)$$

where E_{M1+MoS_2} is the total energy of M_1 (H, Li and Na) adsorbed MoS_2, E_{MoS_2} is the energy of MoS_2 before the adsorption and E_{M1} is the energy of an isolated M_1 atom. According to the definition, the structure with a more negative formation energy is more stable.

3. Supplementary Note 3 - PL data for chemical treated MoS_2 and WS_2

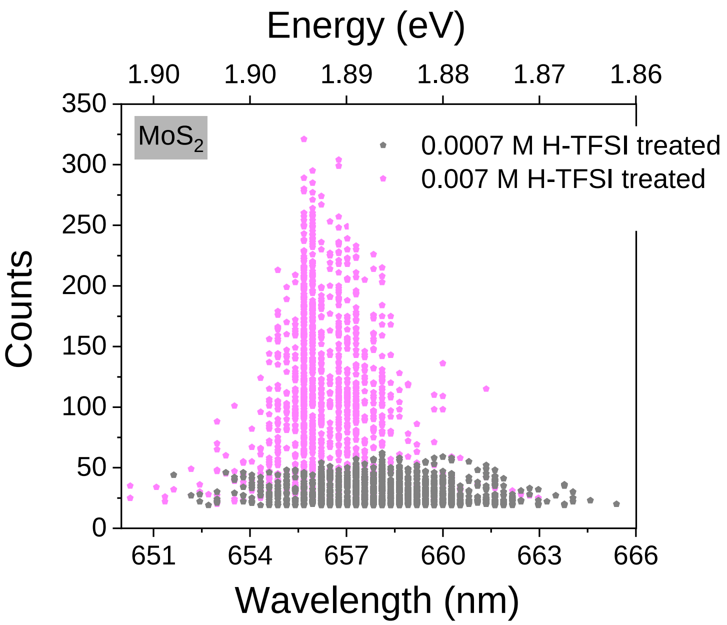

Supplementary Fig. 1 PL enhancement scatter plots of spectral position of the peak emission and peak H-TFSI-treated monolayer MoS_2 PL counts extracted from PL maps of MoS_2 monolayer on Si-SiO_2 (90 nm) after surface treatment with different concentrations of H-TFSI in 1, 2-dichloroethane.

The PL of pristine MoS_2 is usually undetectable due to the low PL intensity, and the corresponding statistic scatter plots of pristine MoS_2 are not presented. As the PL of pristine WS_2 is detectable, the corresponding pristine monolayer scatter plots are also shown in Supplementary Fig. 2. For chemically treated WS_2 samples, we performed PL mapping on the same monolayer as on the pristine sample to obtain more direct observation of the PL enhancing strength of different chemical treatments. The PL of pristine WS_2 is inhomogeneous, and the position of PL maxima varies between 615 nm and 630 nm, which may be due to randomly-distributed disorder potentials, trions, dielectric disorder as well as interactions with optical phonons.[1–3] Upon chemical treatments, the PL of both MoS_2 and WS_2 increase and blue shift statistically, indicating an reduction of trions in both materials. This trend agrees well with previous observations reported by other groups.[4–6]

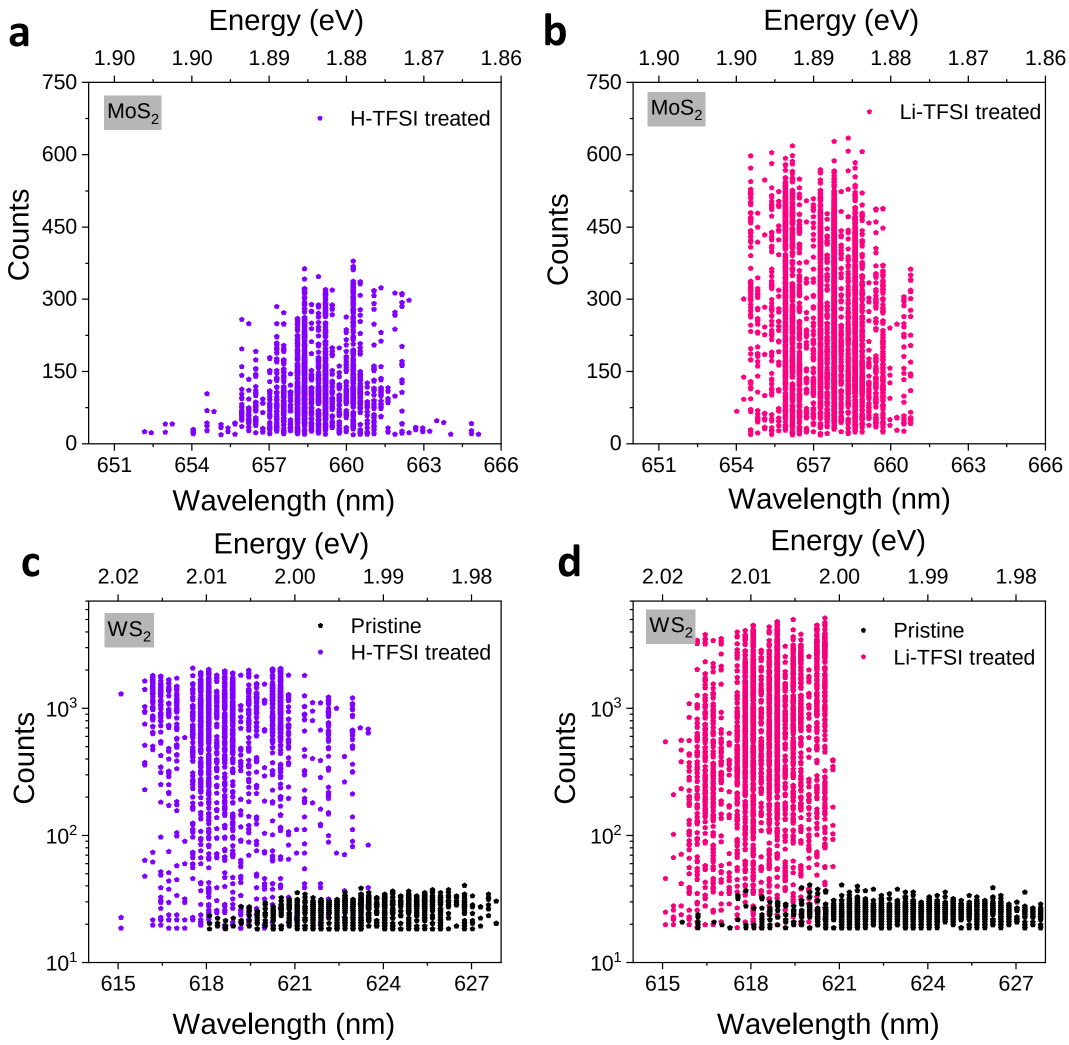

Supplementary Fig. 2 Photoluminescence scatter plots showing **a** peak H-TFSI-treated monolayer MoS_2 PL counts, **b** peak Li-TFSI-treated monolayer MoS_2 PL counts, **c** peak Na-TFSI-treated monolayer MoS_2 PL counts, **d** peak H-TFSI-treated and corresponding pristine monolayer WS_2 PL counts, **e** peak Li-TFSI-treated and corresponding pristine monolayer WS_2 PL counts, and **f** peak Na-TFSI-treated and corresponding pristine monolayer WS_2 PL counts. Data derived from raw spectra from PL maps.

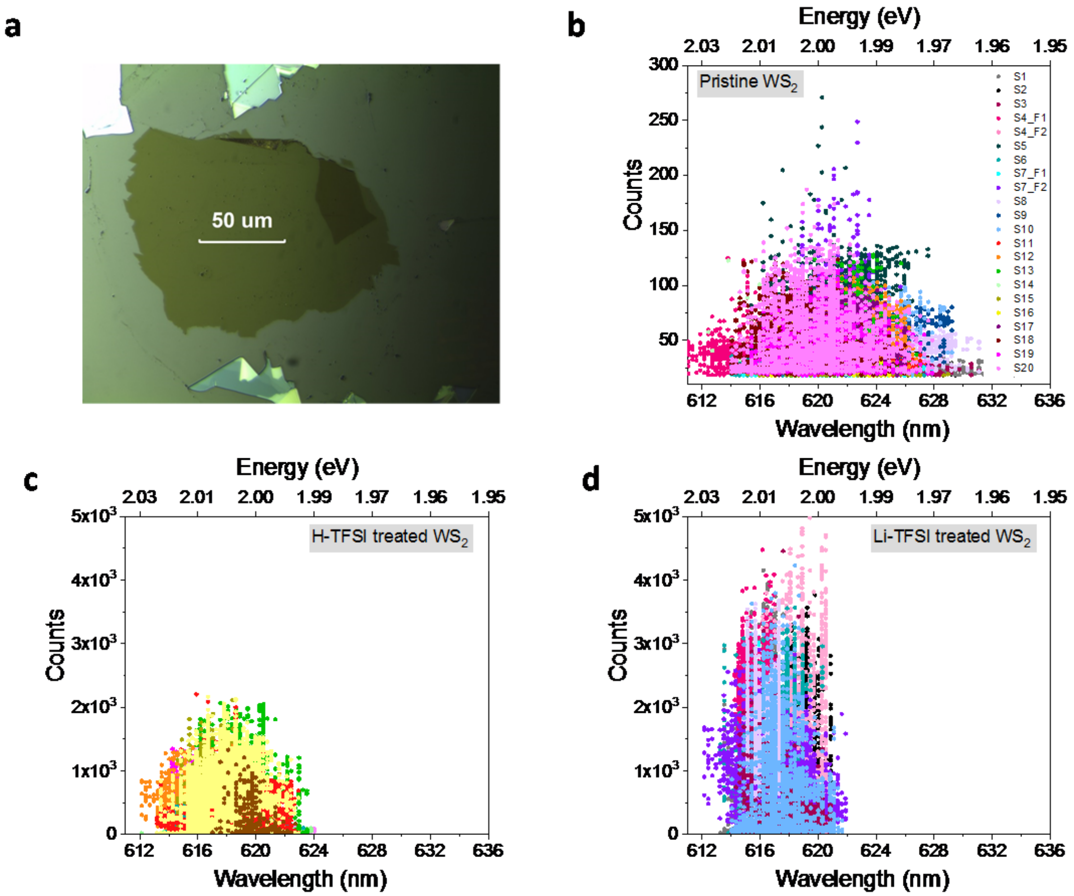

Supplementary Fig. 3 a A typical optical microscope image of mechanically exfoliated WS_2 sample on Si-SiO_2 (90 nm) substrate. **b** PL scatter plots showing peak PL counts of 20 pristine monolayer WS_2 samples (20 samples from sample 1 (S1) to sample 20 (S20) are measured; on sample S4 and Sample S7, two different monolayer flakes (F1 and F2) are measured). **c** PL enhancement scatter plots showing peak PL counts of H-TFSI-treated monolayer WS_2 (10 pristine WS_2 samples from S11 to S20 are treated with H-TFSI). **d** PL enhancement scatter plots showing peak PL counts of Li-TFSI-treated monolayer WS_2 (10 pristine WS_2 samples from S1 to S10 are treated with Li-TFSI).

As shown in Supplementary Fig. 3, 20 different WS_2 monolayer samples are investigated. The monolayer area on each sample varies from 50 to 150 μm to obtain a reasonably big data set. 10 WS_2 monolayer samples are treated with H-TFSI in DCE and the other 10 WS_2 monolayer samples are treated with Li-TFSI in methanol. As shown in Supplementary Fig. 3 b, pristine WS_2 samples are intrinsically doped to different levels and the distribution of PL peak positions from pristine WS_2 samples covers a wide range, varying by more than 60 meV.

The PL intensity of the pristine WS_2 samples also shows huge variations. The high PL intensity can be more than 30 times brighter in comparison with the low ones. On the other hand, after both chemical treatments the PL peak position blueshifts accompanied by a more uniform emission profile. Moreover, the PL intensity is largely enhanced, and in general the PL intensity of Li-TFSI-treated WS_2 samples doubles that of H-TFSI-treated ones despite of the variation of the pristine monolayer properties.

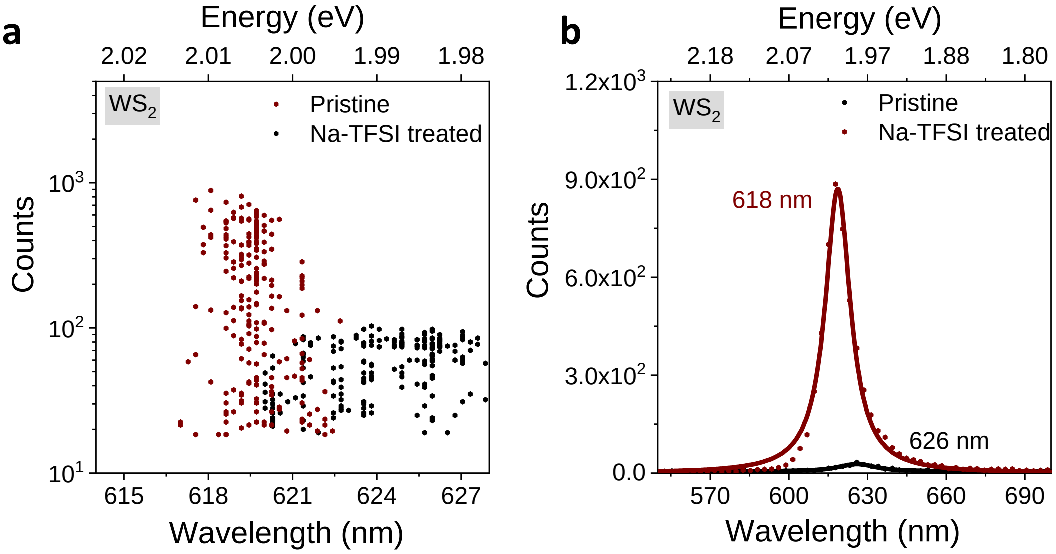

Supplementary Fig. 4 a PL enhancement scatter plots showing peak Na-TFSI-treated monolayer WS_2 PL counts. **b** Maximum PL spectra for pristine and Na-TFSI-treated monolayer WS_2.

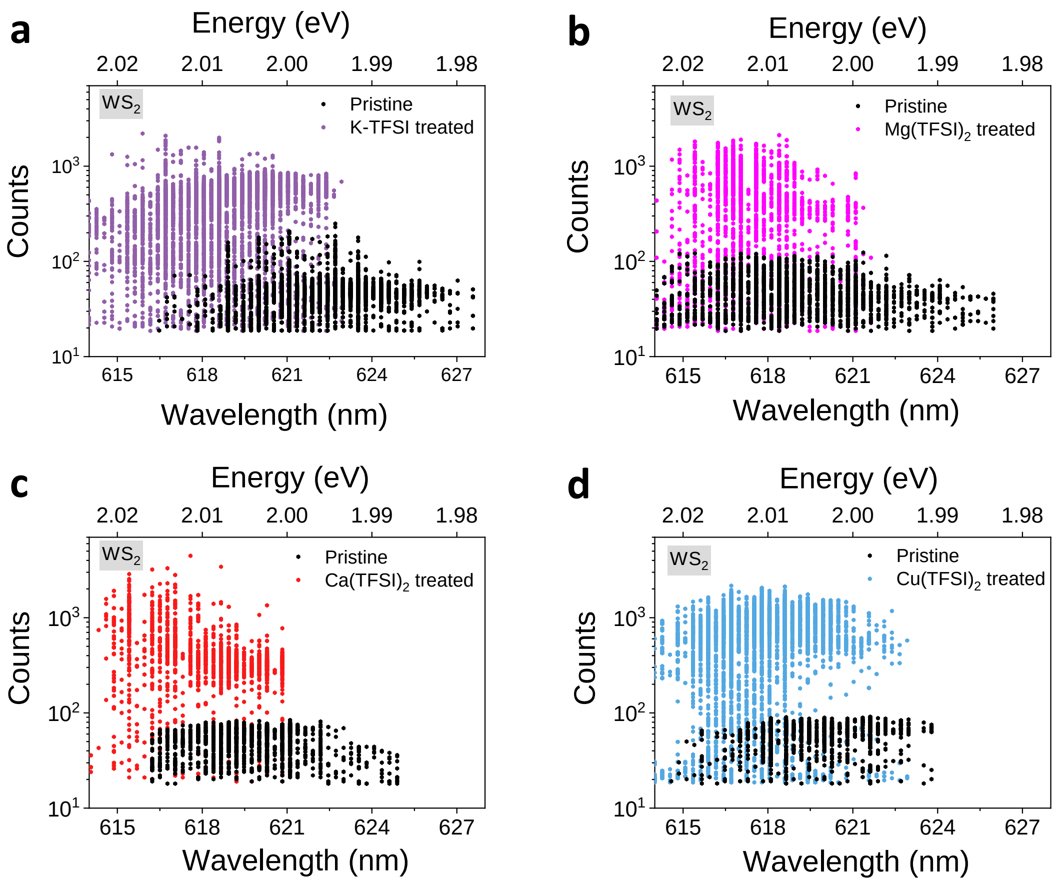

Supplementary Fig. 5 a Chemical structures of K-TFSI and $M_2(TFSI)_2$ (M2 = Mg, Ca and Cu). PL scatter plots showing peak counts of **b** pristine and K-TFSI-treated WS_2, **c** pristine and $Mg(TFSI)_2$-treated WS_2, **d** pristine and $Ca(TFSI)_2$-treated WS_2, and **e** pristine and $Cu(TFSI)_2$-treated WS_2.

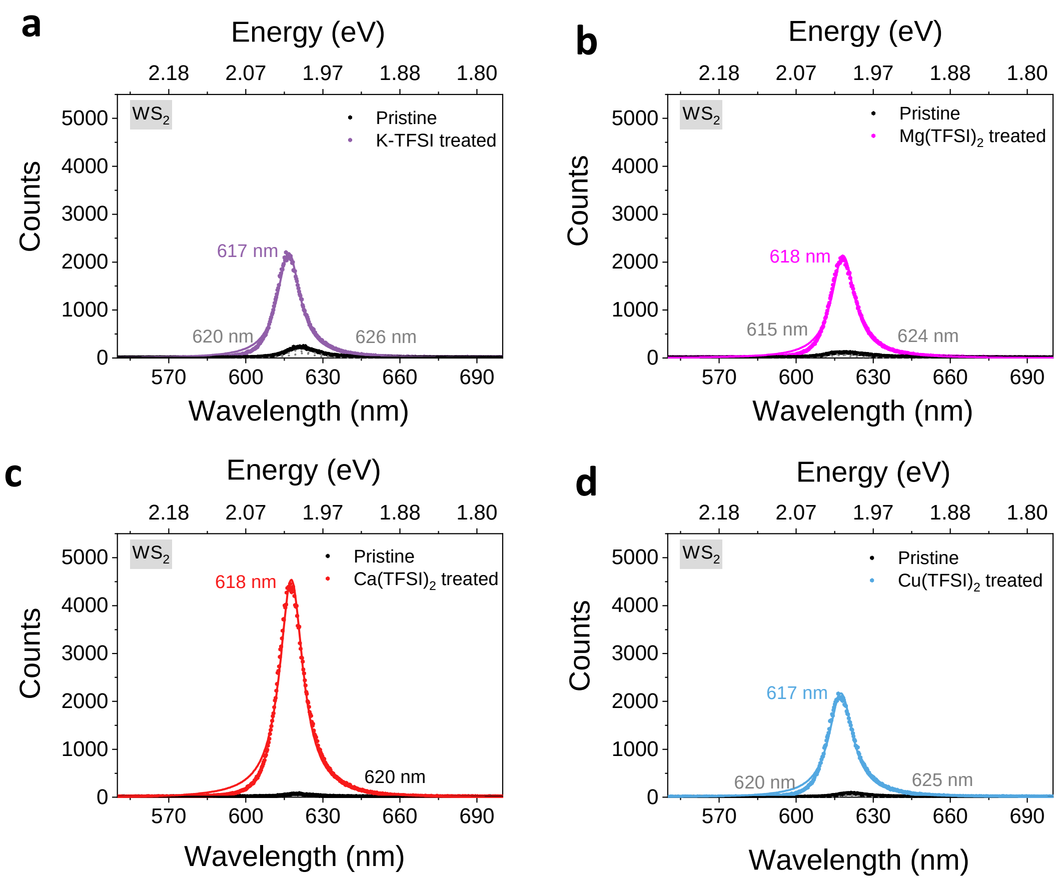

Supplementary Fig. 6 a Maximum PL spectrum for pristine and K-TFSI-treated monolayer WS_2. **b** Maximum PL spectrum for pristine and $Mg(TFSI)_2$-treated monolayer WS_2. **c** Maximum PL spectrum for pristine and $Ca(TFSI)_2$-treated monolayer WS_2. **d** Maximum PL spectrum for pristine and $Cu(TFSI)_2$-treated monolayer WS_2. The decomposed Lorentzian peak fitting is presented in dash line and the cumulative peak fitting is presented in solid line.

To further evaluate if the cationic radii of TFSI salts play a role during the chemical treatments and if other TFSI salts can also enhance PL of TMDSs, we also investigated the effect of other five TFSI based ionic salts on the PL enhancement of WS_2. $Mg(TFSI)_2$ and $Cu(TFSI)_2$ show smaller cationic radii compared to Li-TFSI, while Na-TFSI, K-TFSI and $Ca(TFSI)_2$ show larger cationic radii compared to Li-TFSI.[7] As shown in Supplementary Fig. 2-6, these ionic salts all have positive effect on the PL of WS_2 and the treatments cause blueshift of PL spectra of WS_2. Interestingly, the PL enhancement of WS_2 are similar by K-TFSI, $Mg(TFSI)_2$ and $Ca(TFSI)_2$ treatments, although their cationic radii are quite different. Thus, no concrete relationship between cationic radii and PL tuning strength can be drawn.

In order to gain mechanistic insight to the chemical treatments on TMDS for PL enhancement, we immersed H-TFSI and Li-TFSI treated WS_2 monolayer samples in the solvents DCE and methanol, respectively. After 5h, no obvious PL change is observed. As illustrated in Supplementary Fig. 7, the PL intensity of H-TFSI or Li-TFSI treated monolayer WS_2 sample drops after 24h immersion in the solvents. However, the PL intensity is still much higher than that of pristine WS_2, which suggests that there is a strong interaction between the chemical and WS_2 surface and not all of it can be washed away. In contrast to the PL scatter plots of H-TFSI treated WS_2 after 24h immersion in DCE, where the PL peaks remain blueshifted compared to that of the pristine WS_2 sample, the PL scatter plots of Li-TFSI treated WS_2 show emission from trions in the longer wavelength after 24h immersion in methanol. This is ascribed to the weak alkaline nature of methanol. After 24h immersion in solvents, the PL intensity can be restored to a great extent by conducting the chemical treatment again. This phenomenon suggests there is no chemical reaction involved during the chemical treatment. In addition, the PL enhancement effect of H-TFSI in DCE and HBr in H_2O is also compared to further certify that dissociated H^+ is not the only requirement for large PL improvement (Supplementary Fig. 8). Moreover, we do not observe phenomena that the emission on the edge of monolayers turns brighter than that of central parts even when the monolayers are immersed in the solution with ionic salts for only a few seconds. The relationship between cationic radii and PL tuning strength is not observed, either. Therefore, we rule out the hypothesis that intercalation between the monolayer and substrates may play an important role in enhancing the PL intensity of monolayer TMDSs, which is contradictory to the previous study.[8] Detailed discussion is as follows.

Part of our experimental results and conclusions are contradictory to previously reported results in the literature (ACS Nano 2017, 11, 9390-9396). In that paper, Li-TFSI did not show superior PL enhancement effect on TMD monolayer compared to H-TFSI. That literature also came up with a new mechanism for chemical treatment on general TMD monolayers, where the intercalation of cations between TMD surface and substrate may play an important role in PL enhancement.

We first would like to clarify that the TMD material utilized in the 2017 paper is monolayers grown using chemical vapour deposition (CVD) processes, while the monolayers studied in our work is mechanic exfoliated. One reason for the different results can be the different

nature of defects existing in these two studied samples. The second difference is the concentration of H-TFSI and Li-TFSI is 0.2 mg/mL, and the solvent for Li-TFSI is a 9:1 mixture of 1,2-dichlorobenzene (DCB) and 1,2-dichloroethane (DCE) in the 2017 paper. However, the increase in concentration is proven to enhance the PL intensity of treated TMDSs further in our experiments (Supplementary Fig. 1) and saturates at 0.02 M (5 mg/mL). The concentration in our study is fixed at 0.02 M (5 mg/mL). More importantly, the ionic salts like Li-TFSI shows limited solubility and cannot fully dissociate in DCB or DCE, methanol is, therefore, chosen to be the solvent for Li-TFSI treatment in our study. Furthermore, some phenomena reported in the 2017 paper like PL intensity enhanced but not blueshifted or that enhancement appears only at part of monolayer area close to the edge are not observed in our study. In addition, since we study the same cation from different salts and acids (HBr, Supplementary Fig. 8) and no relationship between cationic radii and PL tuning strength cannot be drawn (discussed in Page SI 8), we can carefully rule out the hypothesis that intercalation between the monolayer surface and substrate play an important role in PL enhancement of TMDSs at the moment.

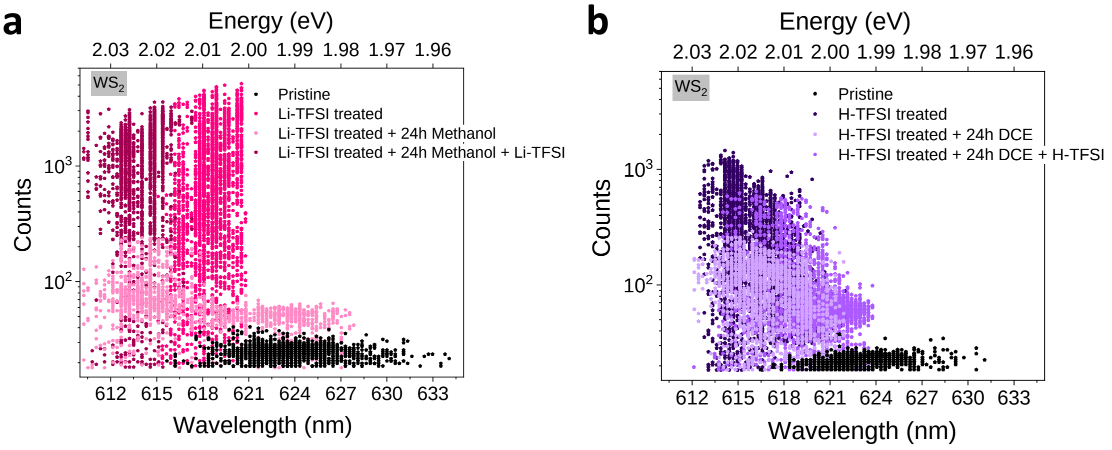

Supplementary Fig. 7 a PL enhancement scatter plots showing peak PL counts of pristine, Li-TFSI-treated, methanol 24 h immersed after Li-TFSI treatment, and Li-TFSI treated after methanol immersion monolayer WS_2. **b** PL enhancement scatter plots showing peak PL counts of pristine, H-TFSI-treated, methanol 24 h immersed after H-TFSI treatment, and H-TFSI treated after methanol immersion monolayer WS_2.

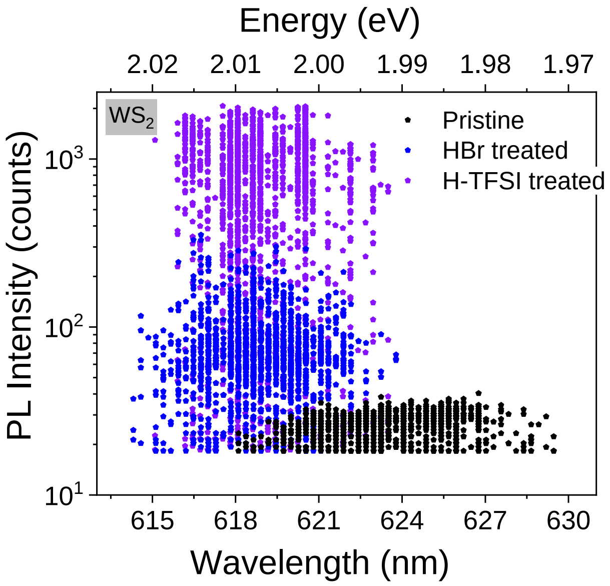

Supplementary Fig. 8 PL enhancement scatter plots showing peak PL counts of pristine, H-TFSI-treated, and HBr-treated monolayer WS_2.

The PL enhancement effect of H-TFSI and HBr is investigated. In our experiment, 0.02 M (5 mg/mL) “super acid” H-TFSI in DCE (pKa = −12) and 47% HBr in H_2O (pKa = − 9) are used. By definition, pKa value tells how much of the acid can actually dissociate, another strong acid HBr is, therefore, chosen in comparison with H-TFSI. Even though H-TFSI possesses higher pKa value, the low concentration leads to lower H^+ concentration compared to acid HBr. The PL spectra of monolayer WS_2 samples blueshift after both H-TFSI and HBr treatment due to p-doping effect (Supplementary Fig. 8). However, the PL intensity of H-TFSI-treated WS_2 samples is nearly 10 times higher than that of HBr-treated sample. This further suggests that previously proposed p-doping effect cannot fully explain the mechanism of chemical treatment on TMDSs.

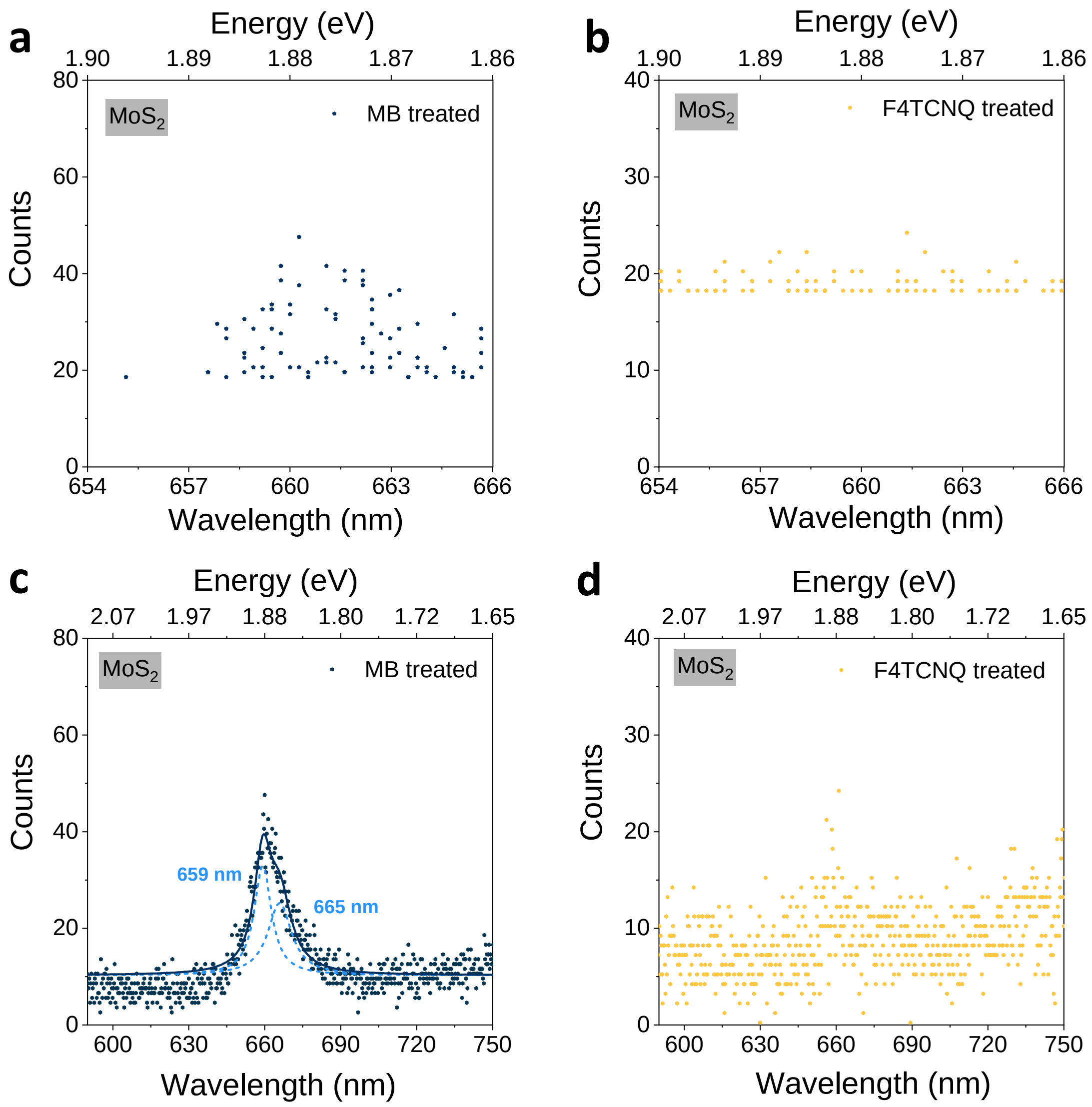

Supplementary Fig. 9 **a** PL enhancement scatter plots showing peak MB-treated monolayer MoS_2 PL counts. **b** PL enhancement scatter plots showing peak F4TCNQ-treated monolayer MoS_2 PL counts. **c** Maximum PL spectrum for MB-treated monolayer MoS_2. **d** Maximum PL spectrum for F4TCNQ-treated monolayer MoS_2. The decomposed Lorentzian peak fitting of MB-treated MoS_2 is presented in dash line and the cumulative peak fitting is presented in solid line.

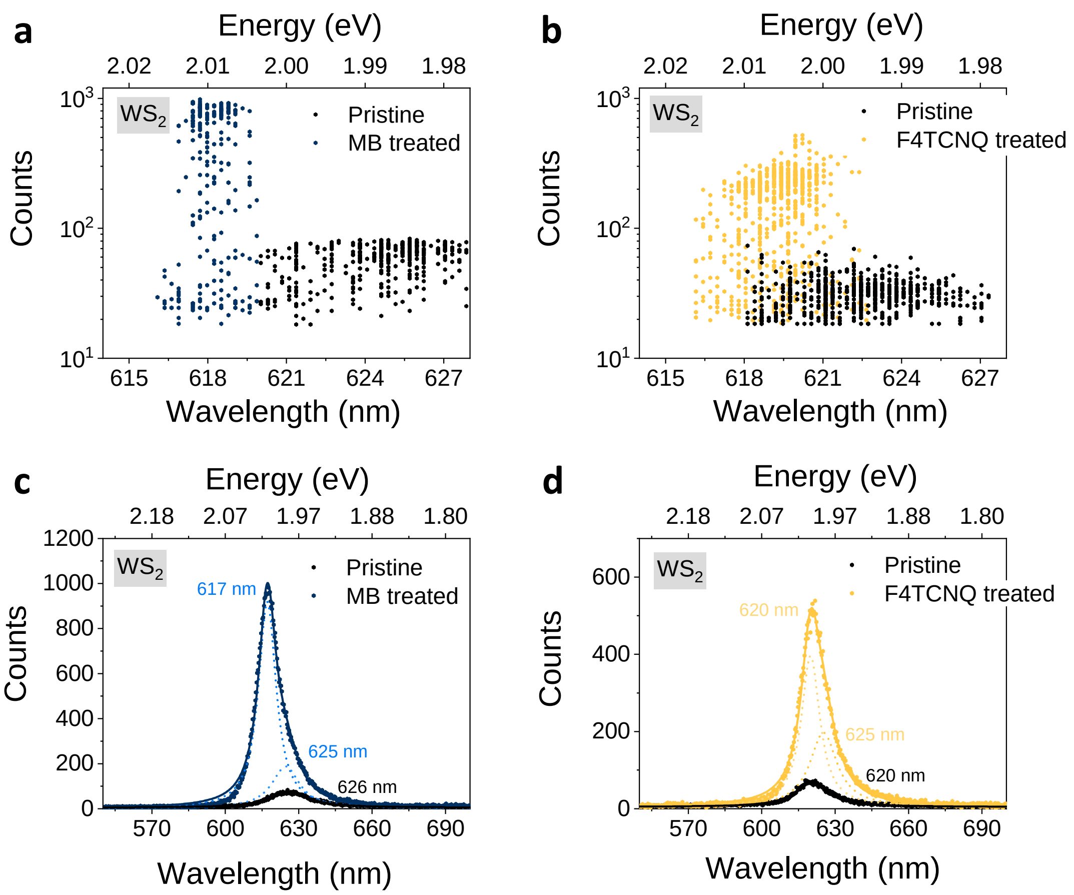

Supplementary Fig. 10 a PL scatter plots showing peak pristine and MB-treated monolayer WS_2 counts. **b** PL scatter plots showing peak pristine and F4TCNQ-treated monolayer WS_2 PL counts. **c** Maximum PL spectrum for MB-treated monolayer WS_2. **d** Maximum PL spectrum for F4TCNQ-treated monolayer WS_2. The decomposed Lorentzian peak fitting of MB and F4TCNQ-treated WS_2 is presented in dash line and the cumulative peak fitting is presented in solid line.

The PL mappings of MB and F4TCNQ-treated WS_2 were performed on the same monolayers as on the pristine samples to obtain more direct observation of the PL enhancing strength of the chemical treatments. As shown in Supplementary Fig. 10, both MB and F4TCNQ increased the PL of WS_2 slightly and blueshifted the PL spectra of WS_2. However, the enhancements are much weaker compared to H-TFSI and Li-TFSI treatments, and there are still clear trion contribution form the emission of MB and F4TCNQ-treated WS_2.

4. Supplementary Note 4 - Raman data for MB and F4TCNQ-treated MoS_2

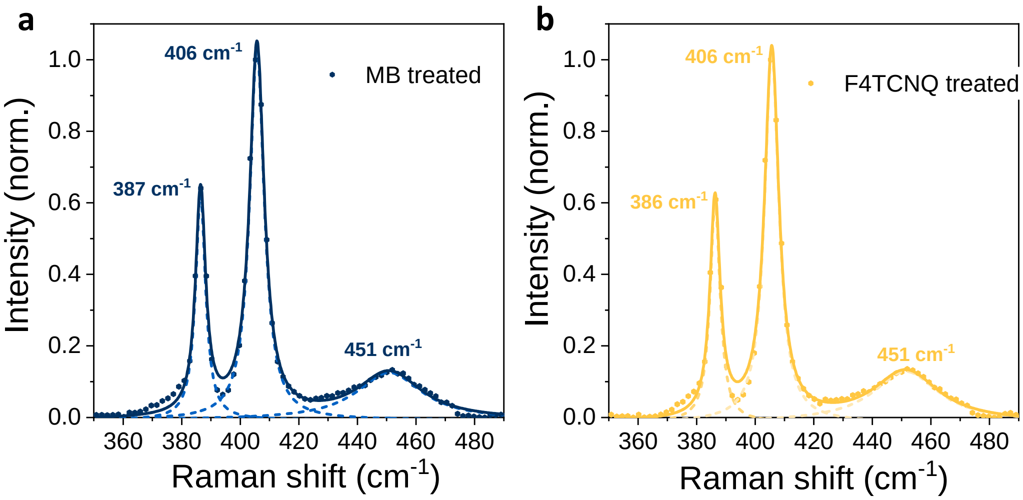

Supplementary Fig. 11 Raman spectra of **a** MB-treated, and **b** F4TCNQ-treated monolayer MoS_2. The decomposed Lorentzian peak fittings of MB and F4TCNQ-treated MoS_2 are presented in dash line and the cumulative peak fittings are presented in solid line.

5. Supplementary Note 5 - XPS data for pristine H-TFSI and Li-TFSI treated MoS_2

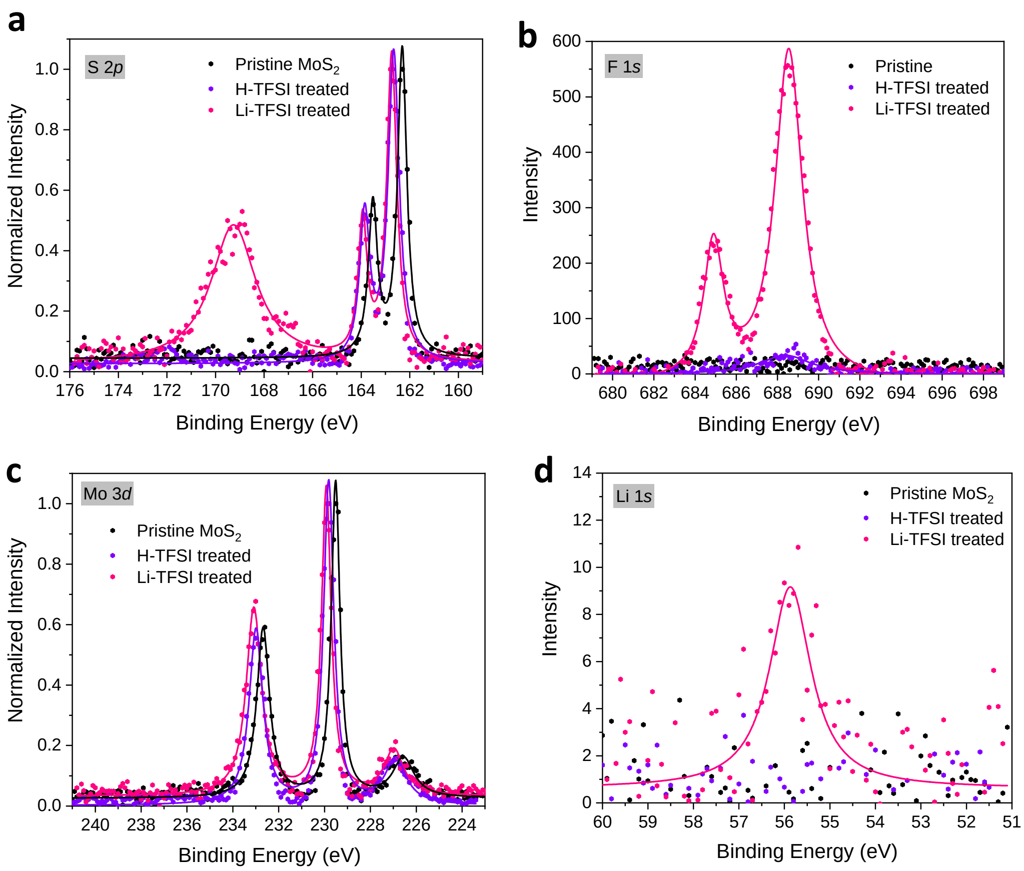

Supplementary Fig. 12 XPS spectra of pristine, H-TFSI-treated, and Li-TFSI-treated monolayer MoS_2. **a** Core level spectra of S 2*p*. **b** Core level spectra of F 1*s*. **c** Core level spectra of Mo 3*d*. **d** Core level spectra of Li 1*s*. The Lorentzian peak fittings of pristine and treated MoS_2 are presented are presented in solid lines.

6. Supplementary Note 6 - DFT simulation data for WS_2

Supplementary Table 1 a DFT simulation of H and Li adsorption energies and the configurations on different positions of monolayer WS_2 surfaces.

	E^{Sv} (eV)	E^{sf} (S) (eV)	E^{sf} (Mo) (eV)	
**H**	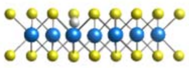	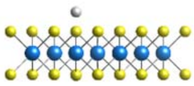	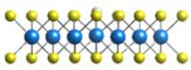	

	-2.29	-0.08	-0.27
**Li**	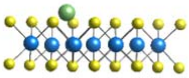 -2.12	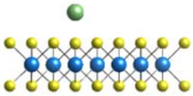 -0.65	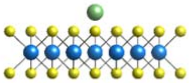 -1.33

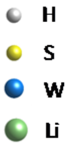

Supplementary Table 1 b DFT simulation of Na, K, Ca and Mg adsorption energies and the configurations on different positions of monolayer WS_2 surfaces.

	E^{Sv} (eV)	E^{sf} (S) (eV)	E^{sf} (W) (eV)
**Na**	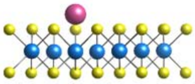 -1.72	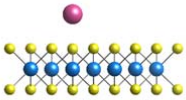 -0.47	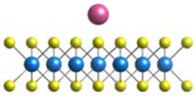 -0.78
**K**	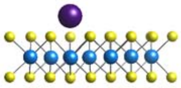 -2.02	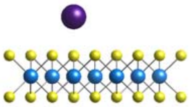 -0.76	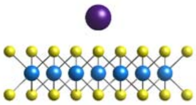 -1.02
**Mg**	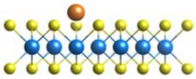 -0.90	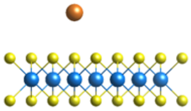 -0.06	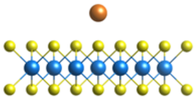 -0.15
**Ca**	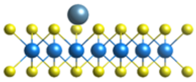 -2.83	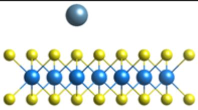 -0.37	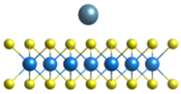 -0.80

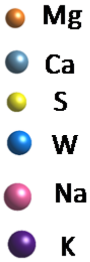

Supplementary Table 1 c DFT simulation of bond energies between M_1^+ and TFSI anion

Bond	Bond Energy (eV)
H-TFSI	4.73
Li-TFSI	5.37
Na-TFSI	4.74
K-TFSI	4.74

As shown in Supplementary Table 1 a, b, similar with MoS_2, all adatoms on WS_2 present a clear preference of adsorption at sulphur vacancy sites compared to the surface of TMDSs. The adsorptions of Li adatom are generally energetically more favourable at surface sites compared to other M_1 atoms. Even though that the adsorptions of M_1 adatoms are energetically more favourable compare to M_2 atoms, each M_2 adatom contributes two positive charges, which explains the effectiveness of M_2TFSI treatments on improving the PL. On the other hands, the effectiveness of M_1-TFSI and M_2TFSI treatments on enhancing PL of TMDSs may also be related to how strongly the cations interact with TFSI anion. This determines the amount of cations interacting with the surfaces of monolayer TMDSs, therefore, the bond energy between cation and TFSI anion is simulated. As shown in Supplementary Table 1 c, all cations present weak interactions with TFSI anion. Moreover, since the solution with ionic salts used during the chemical treatments is dilute and excessive, we assume there are enough cations interacting with the surface of TMDSs in all cases.

7. Supplementary Note 7 - pump-probe and TRPL spectra for MoS_2

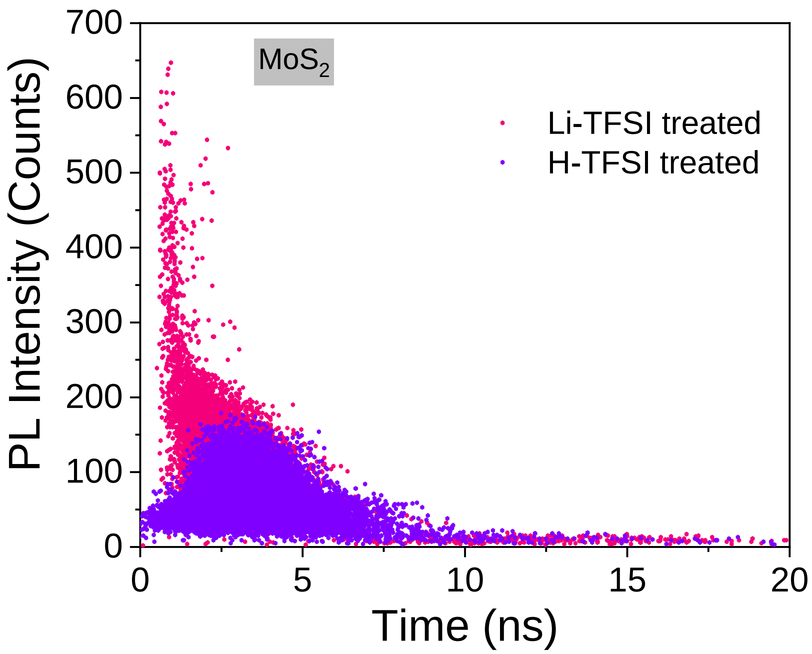

Supplementary Fig. 13 Average lifetime versus PL count for H-TFSI and Li-TFSI treated MoS_2 samples. Each data point plots the average lifetime (x-axis) and PL intensity (y-axis)

for a different spot measured from a 2D map taken on the monolayer flake for each chemical treatment.

Supplementary Table 2. Fitting results for the rates with 15 μJ cm^{-2} in TRPL measurement.

Sample	A_1	τ_1 (ns)	A_2	τ_2 (ns)	A_3	τ_3 (ns)	$<\tau>$ (ps)
H-TFSI treated MoS_2	0.23	0.73	1.25	0.23	0.003	10.00	320
Li-TFSI treated MoS_2	1.78	0.12	0.04	0.77	0.005	6.34	150

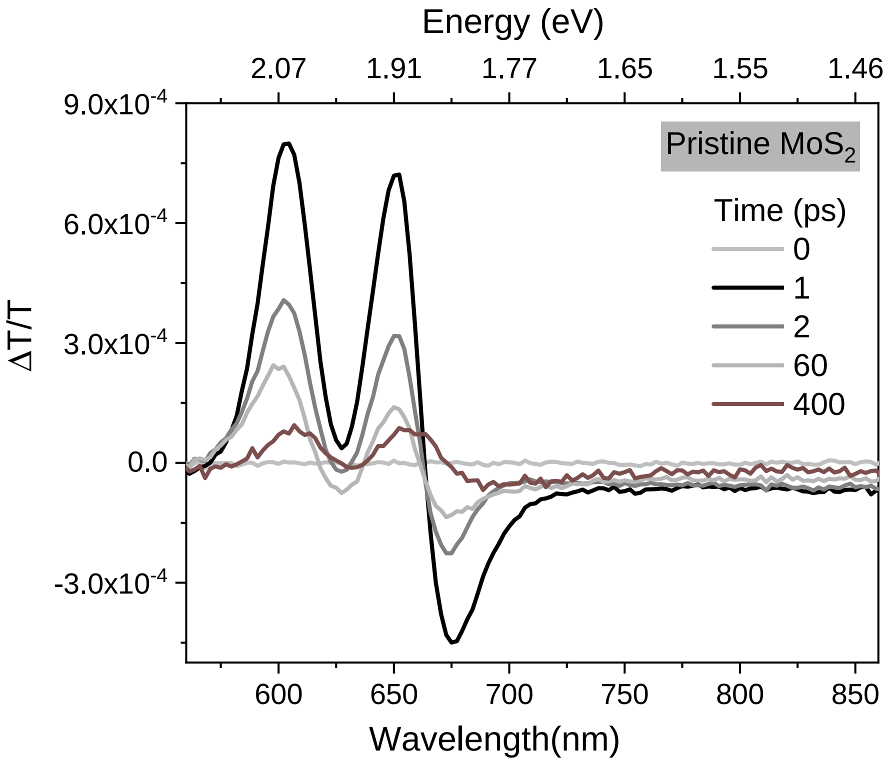

Supplementary Fig. 14 Ultrafast pump-probe data of pristine MoS_2.

8. Supplementary Note 8 - PL data for M_3-Tf and Li-OAc-treated MoS_2 and WS_2

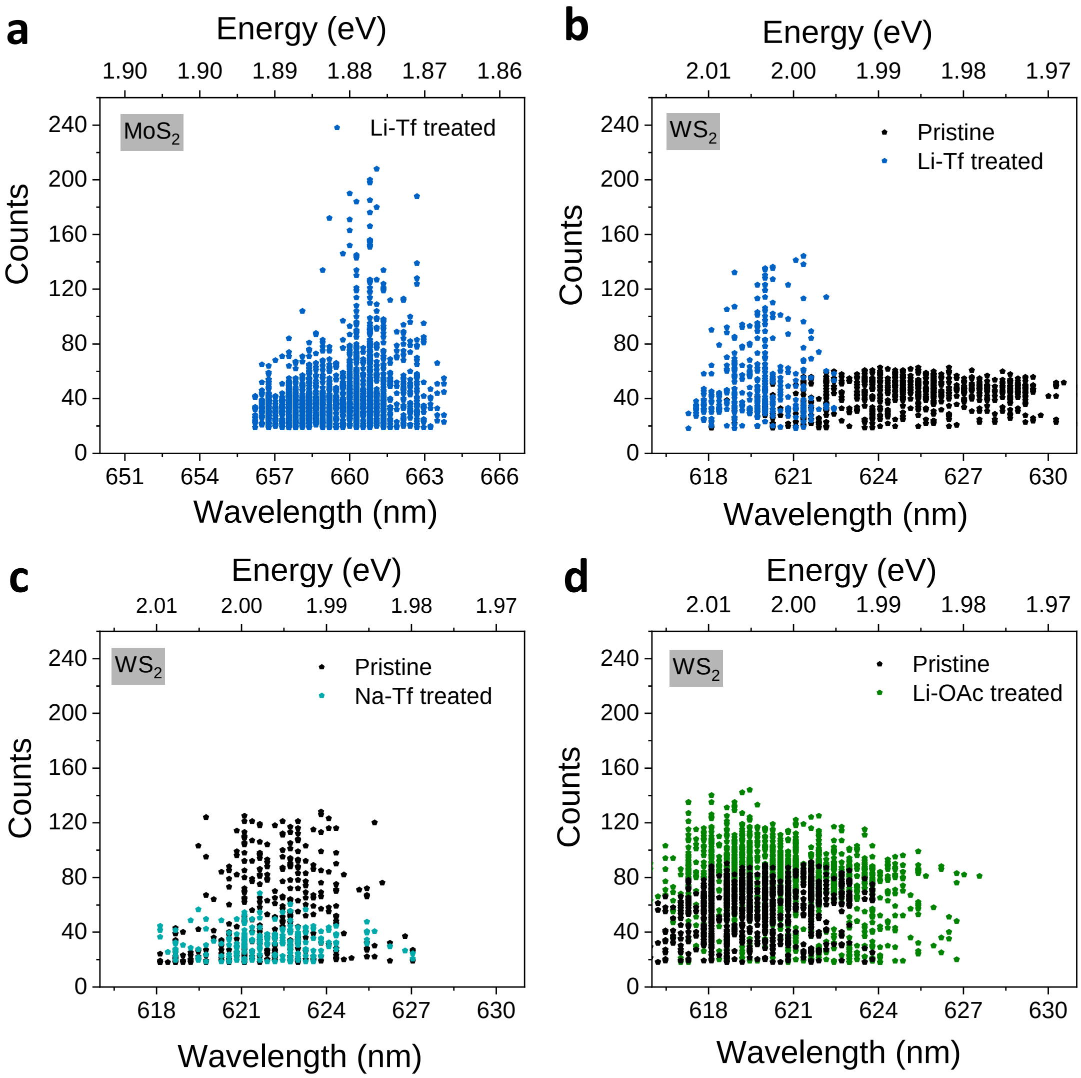

Supplementary Fig. 15 PL enhancement scatter plots showing peak **a** Li-Tf-treated monolayer MoS_2 PL counts, **b** pristine and Li-Tf-treated monolayer WS_2 PL counts, **c** pristine and Na-Tf-treated monolayer WS_2 PL counts, and **d** pristine and Li-OAc-treated monolayer WS_2 PL counts.

9. Supplementary Note 9 - DFT simulation of anion adsorption on MoS_2 surface

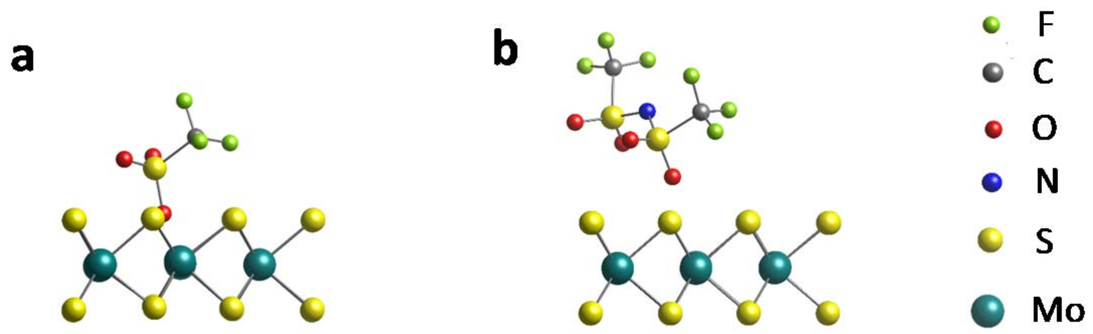

Supplementary Fig. 16 DFT simulation of **a** Tf and **b** TFSI anion adsorption at sulphur vacancy sites of monolayer MoS_2 surfaces.

10. Supplementary Note 10 - Raman data for M_3-Tf and Li-OAc-treated MoS_2

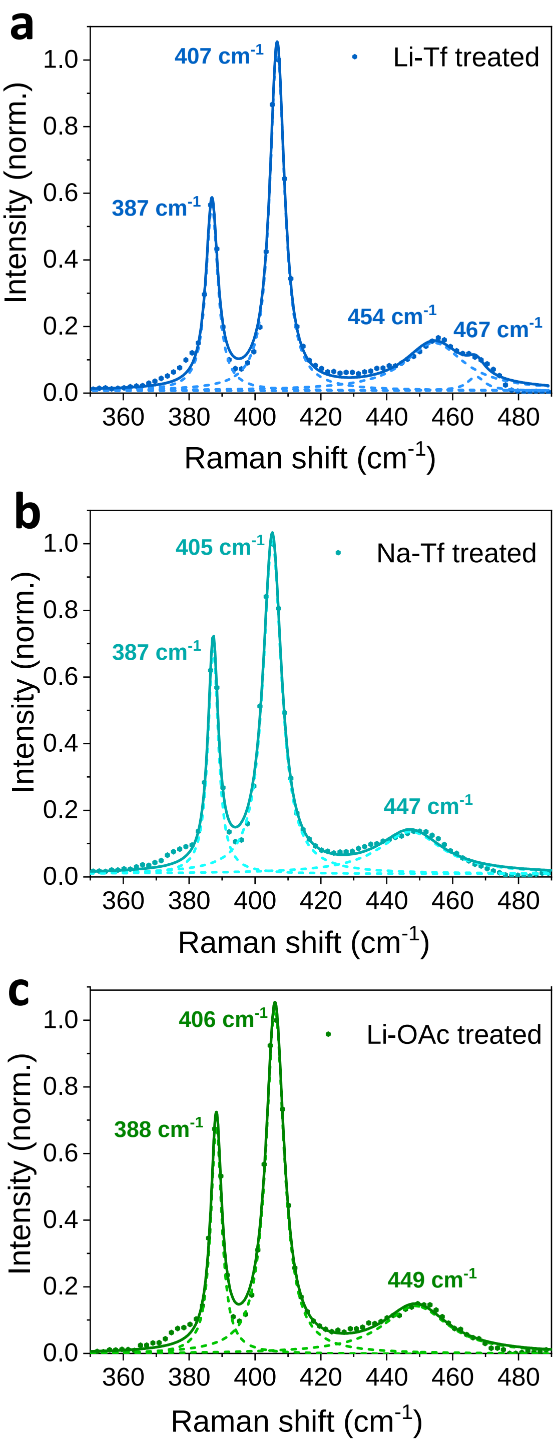

Supplementary Fig. 17 Raman spectra of **a** Li-Tf-treated, **b** Na-Tf-treated, and **c** Li-OAc-treated monolayer MoS_2. The decomposed Lorentzian peak fitting of each spectrum is presented in short dash line and the cumulative fitting is presented in solid line.

11. Supplementary Note 11 - TRPL and PL diffusion data for Li-Tf-treated MoS_2

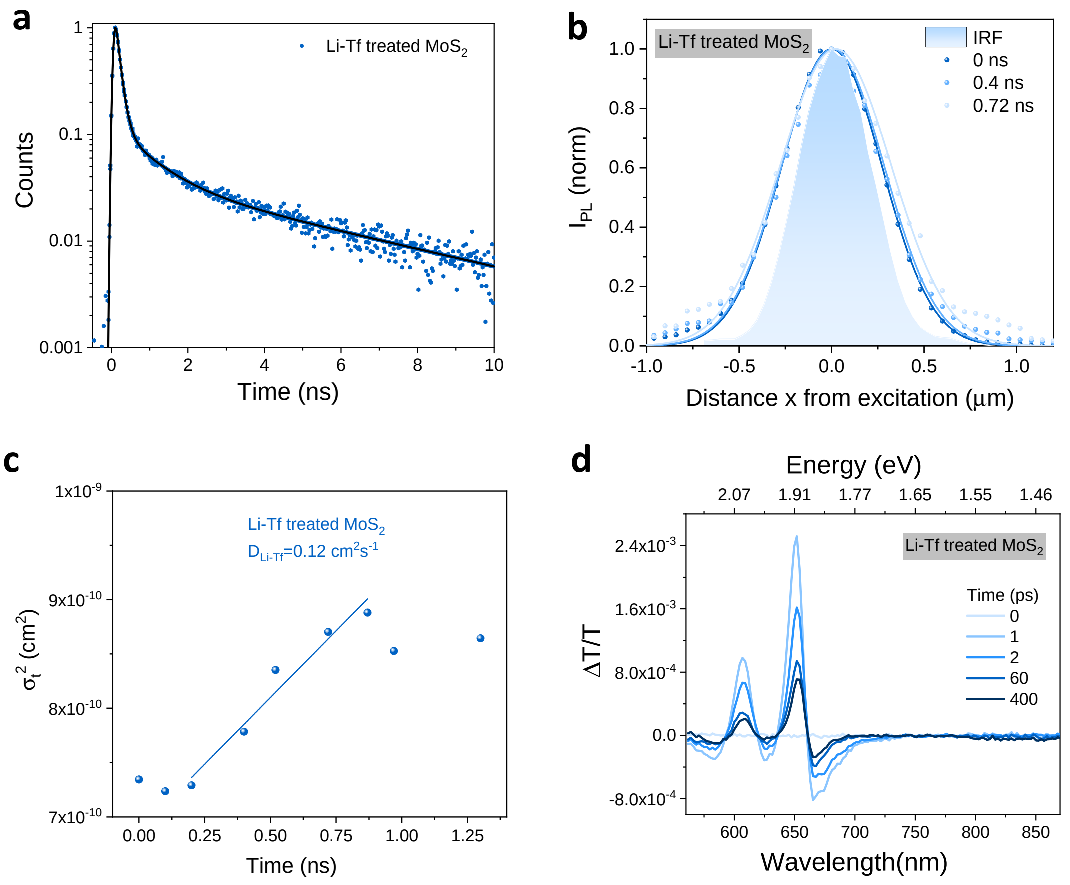

Supplementary Fig. 18 a TRPL decay curve for Li-Tf-treated monolayer MoS_2. **b** Spatial profile of the normalized PL intensity I_{PL} at snapshot t = 0, 0.4 and 0.72 ns for Li-Tf-treated monolayer MoS_2. **c** Corresponding σ_t^2 as a function of time. **d** Ultrafast pump-probe spectra of Li-Tf-treated monolayer MoS_2.

12. References

1. Kash, J. A. Comment on 'origin of the stokes shift: A geometrical model of exciton spectra in 2D semiconductors'. *Phys. Rev. Lett.* **71**, 1286 (1993).
2. Kolesnichenko, P. V., Zhang, Q., Zheng, C., Fuhrer, M. S. & Davis, J. A. Disentangling the effects of doping, strain and defects in monolayer WS2 by optical spectroscopy. (2019) doi:10.1088/2053-1583/ab626a.

3. Raja, A. *et al.* Dielectric disorder in two-dimensional materials. *Nat. Nanotechnol.* **14**, 832–837 (2019).
4. Amani, M. *et al.* Near-unity photoluminescence quantum yield in MoS2. *Science* **350**, 1065–1068 (2015).
5. Lien, D. H. *et al.* Electrical suppression of all nonradiative recombination pathways in monolayer semiconductors. *Science* **364**, 468–471 (2019).
6. Bertolazzi, S., Gobbi, M., Zhao, Y., Backes, C. & Samorì, P. Molecular chemistry approaches for tuning the properties of two-dimensional transition metal dichalcogenides. *Chem. Soc. Rev.* **47**, 6845–6888 (2018).
7. Rahm, M., Hoffmann, R. & Ashcroft, N. W. Atomic and Ionic Radii of Elements 1–96. *Chem. - A Eur. J.* **22**, 14625–14632 (2016).
8. Yu, Y. *et al.* Enhancing Multifunctionalities of Transition-Metal Dichalcogenide Monolayers via Cation Intercalation. *ACS Nano* **11**, 9390–9396 (2017).
